# Matrix Metalloproteinase 7 Is Associated with Symptomatic Lesions and Adverse Events in Patients with Carotid Atherosclerosis

**DOI:** 10.1371/journal.pone.0084935

**Published:** 2014-01-06

**Authors:** Azhar Abbas, Pål Aukrust, David Russell, Kirsten Krohg-Sørensen, Trine Almås, Dorte Bundgaard, Vigdis Bjerkeli, Ellen Lund Sagen, Annika E. Michelsen, Tuva B. Dahl, Sverre Holm, Thor Ueland, Mona Skjelland, Bente Halvorsen

**Affiliations:** 1 Department of Neurology, University of Oslo, Oslo, Norway; 2 Research Institute of Internal Medicine, University of Oslo, Oslo, Norway; 3 Department of Thoracic and Cardiovascular Surgery, University of Oslo, Oslo, Norway; 4 Section of Clinical Immunology and Infectious Diseases, Oslo University Hospital, Rikshospitalet, University of Oslo, Oslo, Norway; 5 Faculty of Medicine, University of Oslo, Oslo, Norway; 6 Department of Neurology, Østfold Hospital Trust, Fredrikstad, Norway; 7 Department of Thoracic and Cardiovascular Surgery, Østfold Hospital Trust, Fredrikstad, Norway; University Heart Center Freiburg, Germany

## Abstract

**Background:**

Atherosclerosis is a major cause of cerebrovascular disease. Matrix metalloproteinases (MMPs) play an important role in matrix degradation within the atherosclerotic lesion leading to plaque destabilization and ischemic stroke. We hypothesized that MMP-7 could be involved in this process.

**Methods:**

Plasma levels of MMP-7 were measured in 182 consecutive patients with moderate (50–69%) or severe (≥70%) internal carotid artery stenosis, and in 23 healthy controls. The mRNA levels of MMP-7 were measured in atherosclerotic carotid plaques with different symptomatology, and based on its localization to macrophages, the *in vitro* regulation of MMP-7 in primary monocytes was examined.

**Results:**

Our major findings were (i) Patients with carotid atherosclerosis had markedly increased plasma levels of MMP-7 compared to healthy controls, with particularly high levels in patients with recent symptoms (i.e., within the last 2 months). (ii) A similar pattern was found within carotid plaques with markedly higher mRNA levels of MMP-7 than in non-atherosclerotic vessels. Particularly high protein levels of MMP-7 levels were found in those with the most recent symptoms. (iii) Immunhistochemistry showed that MMP-7 was localized to macrophages, and *in vitro* studies in primary monocytes showed that the inflammatory cytokine tumor necrosis factor-α in combination with hypoxia and oxidized LDL markedly increased MMP-7 expression. (iv) During the follow-up of patients with carotid atherosclerosis, high plasma levels of MMP-7 were independently associated with total mortality.

**Conclusion:**

Our findings suggest that MMP-7 could contribute to plaque instability in carotid atherosclerosis, potentially involving macrophage-related mechanisms.

## Introduction

Atherosclerosis is a major cause of cerebrovascular and coronary heart disease. It is now well established that inflammation plays an important role in the pathogenesis of this chronic and progressive disease at least partly though its bidirectional interaction with lipids [1]. Matrix remodeling is another important feature of atherosclerosis [2], [3]. The formation of a fibrous cap is an important step in atherogenesis that involves the interaction between collagen producing vascular smooth muscle cells (SMC) and infiltrating immune cells such as T cells and monocytes [2], [3]. While enhanced fibrogenesis may narrow the lumen and potentially result in chronic ischemia, it may also stabilize the lesion, preventing plaque rupture with subsequent thrombus formation and adverse clinical events (e.g., transient ischemic attack [TIA], stroke and myocardial infarction [MI]) [4].

It seems that the strength of the fibrous atherosclerotic cap depends on a dynamic balance between collagen synthesis and degradation. The activity of matrix metalloproteinases (MMPs) is of major importance for matrix degradation within an atherosclerotic plaque, potentially turning the lesion from a stable to an unstable phenotype [5]. MMPs are a group of proteinases consisting of at least 23 structurally related members that degrade fibrillar collagen type I and III, proteoglycans, collagen, and elastin, which are all substantial constituents of the fibrous atherosclerotic cap [6]. Inflammatory cytokines may increase the production of MMPs in macrophages and vascular SMC. In addition, MMPs may enhance the bioactivity of some inflammatory cytokines (e.g., tumor necrosis factor [TNF]-α and interleukin [IL]-1β) by proteolytic cleavage, representing a pathogenic inflammatory loop in atherogenesis.

There are several reports of increased levels of MMPs such as MMP-1, MMP-2, MMP3, MMP-8, MMP-9, and MMP-12 in patients with atherosclerotic disorders [7]–[9]. MMP-7 has been linked to apoptosis of vascular SMC, and in contrast to several other MMPs, has also been associated with foam cells along the necrotic core of the lesion, suggesting an important role in plaque destabilization [10]. However, at present, there are relatively few studies on MMP-7 in clinical atherosclerosis. In a small population (n = 8), using cDNA microarray, we identified MMP-7 as one of 87 genes that was markedly up-regulated in symptomatic carotid plaques (see online Supplemental file in reference [11]). To further elucidate the role of MMP-7 in atherosclerosis and plaque destabilization, we examined MMP-7 levels in patients with carotid plaque in relation to symptomatology, both systemically and within the lesion, and plasma levels of MMP-7 were also related to mortality during follow-up. In addition, we analyzed the regulation of MMP-7 in primary monocytes in response to stimuli with relevance to atherogenesis such as inflammatory cytokines and lipids.

## Materials and Methods

### Patients and Controls

One hundred and eighty-two consecutive patients, with moderate (50–69%) or severe (≥70%) internal carotid artery stenosis, treated with carotid endarterectomy (n = 149) or carotid artery stenting (n = 10) or conservatively (n = 23) were recruited at Department of Neurology, Oslo University Hospital Rikshospitalet (Table 1). The patients were classified into two groups according to their symptoms: (i) 73 (40%) patients had a stroke, TIA, or amaurosis fugax ipsilateral to the stenotic internal carotid artery in the previous 2 months, and (ii) 109 patients (60%) patients had symptoms >2 months ago or no relevant symptoms. Asymptomatic carotid stenosis was detected during clinical examinations of patients with coronary artery disease, peripheral artery disease or stroke/TIA more than 6 months ago. The carotid stenosis was diagnosed and classified by precerebral color duplex and CT angiography according to consensus criteria [12]. Ultrasound plaque appearance in terms of echogenisity was classified according to consensus criteria [13]. Exclusion criteria were severe concomitant disease such as infections, connective tissue disease, or malignancies, heart failure, and liver or kidney disease. For comparison, blood samples were also collected from 23 healthy control subjects (age 58.3, SD 6.8 years; 15 males; body mass index 24.5, SD 2.6 kg/m^2^; creatinine72.5, SD 10.8 µmol/L; LDL cholesterol 1.8, SD 0.4 mmol/L; HDL cholesterol 1.6, SD 0.3 mmol/L; triglycerides 1.0 SD, 0.5 mmol/L; HbA1C 5.0, SD 0.5%). The controls were recruited from the same area of Norway as the patients. All controls subjects were healthy individuals as assessed by disease history, clinical examination, and normal levels of high sensitivity C-reactive protein (CRP). The protocols were approved by the Regional Committee for Medical and Research Ethics, South-East, Norway, ref S-0923a 2009/6065. The study confirms with the principles outlined in the Declaration of Helsinki for use of human tissue or subjects. Signed informed consent for participation in the study was obtained from all individuals.

**Table 1:** Baseline variables in patient groups according to symptomatology (n = 182).

	Symptomatic	Asymptomatic	
	(symptoms <2 months )	(symptoms ≥2 months or no symptoms)	p
	n = 73	n = 109	
Age, year*	66.9 (8.7)	66.5 (8.7)	0.93
Male sex, % (n)	65.8 (48)	63.3 (69)	0.73
Diabetes mellitus, % (n)	17.8 (13)	17.4 (19)	0.95
Coronary disease, % (n)	47.9 (35)	47 (49)	0.65
Statin treatment, % (n)	87.7 (64)	84.4 (92)	0.5
Acetylsalisylic acid, % (n)	86.3 (63)	80.7 (88)	0.3
Clopidogrel, % (n)	46.6 (34)	21.1 (23)	0
Dipyramidole, % (n)	11 (8)	15.6 (17)	0.26
Warfarin, % (n)	11 (8)	12 (13)	0.83
Hypertension, % (n)	67.1 (49)	67.9 (74)	0.94
Ipsilateral ischemia on cerebral MRI, % (n)	65,8 (48)	52.3 (57)	0.08
Echolucent plaque, % (n)	34.2 (25)	27.5 (30)	0.07
Degree of stenoses, % **	80 (50–99)	80 (50–99)	0.2
Nicotin, % (n)	43.4 (39)	51.4 (56)	0.98
MMP-7, ng/ml**	2.32 (0.13–10.95)	1.83 (0.33–14.88)	**0.048**
BMI, per kg/m^2^*	25.9 (4.3)	26.2 (3.8)	0.42
Cholesterol, mmol/l*	4.4 (1.1)	4.3 (0.8)	0.81
HDL cholesterol, mmol/l*	1.3 (0.5)	1.3 (0.4)	0.48
LDL cholesterol, mmol/l*	2.7 (1.0)	2.5 (0.7)	0.73
Triglycerides, mmol/l*	1.4 (0.7)	1.5 (0.8)	0.71
CRP, mg/l*	5.5 (6.8)	5.5 (7.5)	0.72
Leukocyte count, 10^9^/l*	7.9 (1.9)	7.7 (2.1)	0.36
Fibrinogen, g/l*	4.0 (0.80)	4.0 (0.96)	0.82
Platelets, 10^9^/l*	286 (76.6)	277 (68.6)	0.3
HbA1c,%*	6.0 (1.2)	6.0 (1.4)	0.66

Clinical symptoms include stroke, TIA or amaurosis fugax ipsilateral to the stenotic internal carotid artery. BMI, body mass index. Numbers are given as percentage (numbers), *mean (SD), or **median (min–max). Cholesterol, HDL, LDL and Triglycerides levels were obtained in respectively 122, 110, 109, and 118 patients.

### Blood Sampling Protocol

Venipuncture of a forearm vein was performed within 2 days before carotid endarterectomy/carotid artery stenting with minimal stasis. Peripheral venous blood was drawn into pyrogen-free tubes with ethylenediaminetetraacetic acid as anticoagulant. The tubes were immediately immersed in melting ice and centrifuged within 30 minutes at 2500 *g* for 20 minutes to obtain platelet-poor plasma. All samples were stored at −80°C and thawed only once.

### Measurement of MMP-7

MMP-7 was analyzed in plasma by Multiplex suspension technology with MMP-7 multiplexable beads from R&D Systems (Minneapolis, MN). The samples were analyzed on a Multiplex Analyser (BioRad, Hercules, CA), and quantification was accomplished by using the BioPlex Manager Software (BioRad). MMP-7 levels in plaque lysates were assessed by an enzyme immunoassay (EIA) from R&D Systems.

### Tissue Sampling from Carotid Plaque and Non-atherosclerotic Vessels

Atherosclerotic carotid plaques were retrieved from patients during carotid endarterectomy. Plaques that were used for RNA and protein extraction were rapidly frozen in liquid nitrogen. Plaques that were used for imunohistochemistry and related-analyses were put in 4% phosphate buffered-formalin for 48 hours and then embedded in paraffin. For comparison, non-atherosclerotic vessels were obtained from the common iliac artery of organ donors (for mRNA expression) and from carotis artery during autopsy (for immunohistochemistry). Control tissues were prepared and stored in the same way as carotid plaques.

### Immunohistochemistry

Sections (5 µm) of paraffin embedded atherosclerotic and non-atherosclerotic vessels were treated with 0.5% H_2_0_2_, followed by high-temperature unmasking in citrate-buffer (pH 6), blocked with 0.5% bovine serum albumin (BSA) and then incubated with primary antibody (rabbit anti-human MMP-7; Abcam, Cambridge, UK) for one hour at room temperature. After washing, the slides were incubated for 30 minutes with peroxidase-conjugated secondary antibodies (Impress-Vector, Vector laboratories, Burlingame, CA), rinsed and developed with chromogen for immunoperoxidase staining (DAB Plus, Vector laboratories) for 7 minutes. The sections were counterstained with Hematoxylin. Omission of the primary antibody served as a negative control.

### Immunfluorescence

Paraffin-embedded sections (5 µm) of atherosclerotic carotid plaques were exposed to high-temperature unmasking (citrate-buffer, pH 6), blocked in 0.5% BSA and incubated over night at 4°C with rabbit anti-human MMP7 (Abcam) and mouse anti-human CD68 (Dako, Glostrup, Denmark). The sections were counterstained with Alexa Fluor 488-conjugated goat anti-rabbit IgG and Alexa Fluor 633-conjugated donkey anti-mouse IgG (both from Invitrogen, Eugene, OR). Nuclei were stained with diamidino-2-phenylindole (DAPI) (Slow Fade Gold antifade reagent, Invitrogen). Fluorescent images were obtained on a Nikon Eclipse E400 microscope with 400× magnification.

### Sirius Red Staining

Paraffin sections of human atherosclerotic plaques were de-waxed, hydrated and stained with Weigerts haematoxylin (Sigma, St Louis, MO) for 8 minutes prior to staining with Picro Sirius Red (Sigma; 0.1% in saturated picric acid solution) for 1 hour. Sections were washed in acidified water, dehydrated and mounted in eukitt. Pictures were obtained in a microscope with circularly polarized light.

### Atherosclerotic Plaque Lysate

Proteins were extracted from atherosclerotic plaques as follows; the tissue powders from the plaques were homogenized in ice-cold lysis buffer (PBS containing protease inhibitor cocktail [GIBCO, Paisley, UK] with 1% Triton X-100 and 0.1% Tween 20) at a ratio of 0.1 mL per 10 mg wet wt tissue by a metal blade homogenizer. Extracts were incubated on ice for 15 minutes and centrifuged at 12,000 *g* (15 minutes at 4°C). The supernatants were retained an d stored at −80°C until further analyses. Protein concentrations in the samples were measured with the bicinchoninic acid method (Pierce, Cheshire, UK).

### Real-time Quantitative RT-PCR

Total RNA was extracted from peripheral blood mononuclear cells (PBMC), primary monocytes and atherosclerotic plaques and non-atherosclerotic vessels using RNeasy spin columns (QIAGEN, Hilden, Germany), subjected to DNase I treatment, and stored at −80°C until further analysis. cDNA was synthesized using high-capacity cDNA archive kits (Applied Biosystems, Foster City, CA). Gene expression was assessed using TaqMan assays; MMP-7: Assay ID Hs01042796_m1, CD45: AssayID Hs00365634_g1 and CD68 AssayID: Hs00154355_m1 (Applied Biosystems) on the ABI Prism 7500 instrument (Applied Biosystems). Gene expression of the reference gene β-actin was used for normalization.

### Isolation and Culturing of Cells

PBMC were obtained from heparinized blood by Isopaque-Ficoll (Lympoptrp; Axis Shield, Oslo, Norway) and stored in liquid nitrogen or used immediately for further isolation of monocytes by monodisperse immunomagnetic beads (Dynal, Oslo, Norway) [14]. Freshly isolated monocytes were cultured for 2 days in RPMI 1640 (PAA laboratories, Pasching, Austria), supplemented with 10% fetal bovine serum, before further incubation for 18 hours with or without endotoxin-free oxidized low-density lipoprotein (oxLDL, 20 µg/ml) from human plasma, prepared and oxidatively modified by Cu^2+^-iones as previously described [15], in combination with TNFα (5 ng/ml, R&D Systems) in either a humidified atmosphere with 5% CO_2_ and 95% air or in hypoxia (5% CO_2_ and 1% O_2_ in a mini Galaxy A hypoxia-chamber; RS Biotech, Irvine, UK).

### Statistical Analyses

For comparison of 2 groups of individuals, the Mann-Whitney U test was used. If more than two groups were groups were compared, the Kruskal-Wallis test was used. If significant, the Mann-Whitney U test was used to assess differences between each pair of groups. The Chi-square test was used for analyzing contingency data. In the *in vitro* studies, Student’s t test (primary monocytes) or Mann-Whitney U test (plaque samples) was used. Coefficient of correlation was calculated by the Pearson or Spearman rank test depending on the distribution of data. Kaplan–Meier analysis with log-rank test was performed to compare the number of events in relation to dichotomized MMP-7 levels. MMP-7 was dichotomized according to the median value of MMP-7 in the patient group as a whole (1.96 ng/mL). The importance of MMP-7 as a risk factor for all-cause mortality was further investigated by multivariable cox-regression including variables that were imbalanced (p<0.05) between survivors and non-survivors (i.e., age, hypertension, recent ischemia on cerebral MRI, and CRP). Due to non-normal distribution of CRP and MMP-7 these were log transformed prior to regression and the Hazard Ratio (HR) is expressed per SD change for these variables. Probability values (2-sided) were considered significant at p<0.05. All calculations were carried out with SPSS for windows statistical software (Version 18.0; SPSS Inc, Chicago, IL).

## Results

### Plasma Levels of MMP-7 in Patients with Carotid Plaques and in Healthy Controls

**Figure 1:**
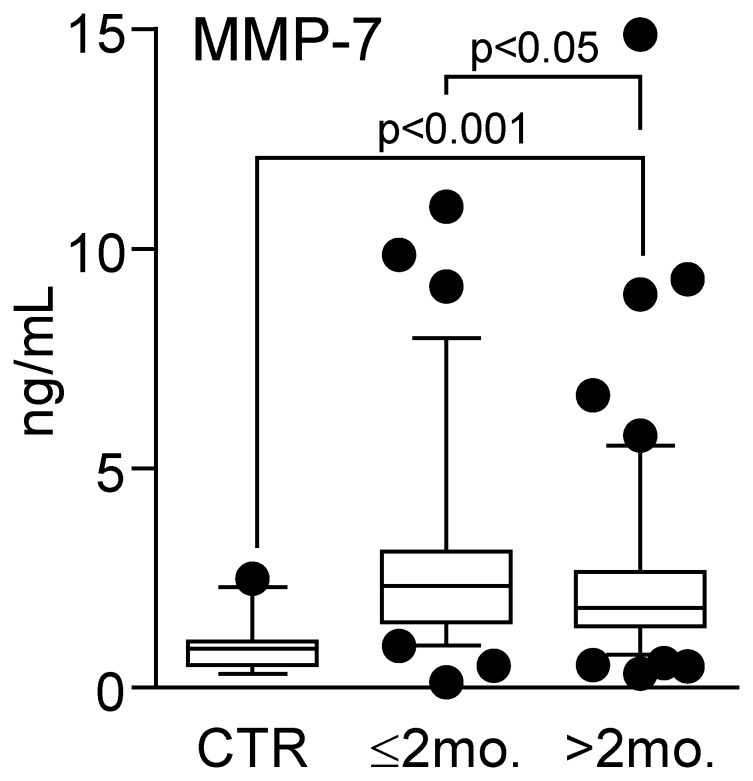
Plasma levels of MMP-7 in patients with carotid plaques in relation to symptomatology. The figure shows plasma levels of MMP-7 in patients with carotid atherosclerosis (n = 182) divided into (i) patients with the most recent symptoms (within the last 2 months, n = 73) and (ii) patients with symptoms >2 months ago and asymptomatic patients (>2 months, n = 109). For comparison, MMP-7 levels were also analyzed in 23 healthy controls (CTR). Data are shown as a box and whisker plot with median (Q1, Q3) in the box and the whiskers representing the 5 and 95 percentiles. Note that both groups of patients have significantly raised MMP-7 levels as compared with controls.

Patients with carotid atherosclerosis (n = 182) had markedly raised plasma levels of MMP-7 compared with healthy controls (n = 23) (1.96 ng/ml versus 0.89 ng/ml, medians; p<0.001). Particularly high levels were found in patients with the most recent symptoms (within the last 2 months), showing significantly higher MMP-7 levels than the other patients (i.e., asymptomatic patients and patients with symptoms more than two months ago) (Fig. 1). In fact, of the parameters that are outlined in Table 1, only MMP-7 was significantly associated with recent ischemic symptoms (i.e., within 2 months). In the patient group as a whole, plasma levels of MMP-7 were *positively* correlated with markers of inflammation (CRP, r = 0.15; p = 0.05 and fibrinogen, r = 0.34; p = 0.005), hypertension (r = 0.20; p = 0.007), triglycerides (r = 0.21; p<0.05) and the presence of recent ischemia on cerebral MRI (r = 0.24; p = 0.005), and *inversely* correlated with HDL cholesterol (r = −0.20; p<0.05). In contrast, there was no correlation between plasma level of MMP-7 and the degree of carotid artery stenosis (r = 0.14, p = 0.23).

We found no association between the use of statins (or any of the other medications that are outlined in Table 1) and MMP-7 levels, and for statins, this may reflect that most of the patients (86%) were using these medications. It has been shown, however, that statins may reduce MMP-7 levels [16], but this will result in an underestimation rather than an overestimation of the difference between patients and healthy controls. The patients were significantly older than the controls (66.6, SD 8.6 years versus 58.3, SD 6.8 years, p<0.001), and MMP-7 was significantly correlated with age in both patients (r = 0.22, p = 0.003) and controls (r = 0.41, p = 0.052), although the correlation in healthy controls did not reach statistical significance. Importantly, however, plasma levels of MMP-7 were significantly raised in patients with carotid atherosclerosis as compared with healthy controls also after adjusting for age (p<0.001).

### The Expression of MMP-7 within Carotid Atherosclerosis

**Figure 2:**
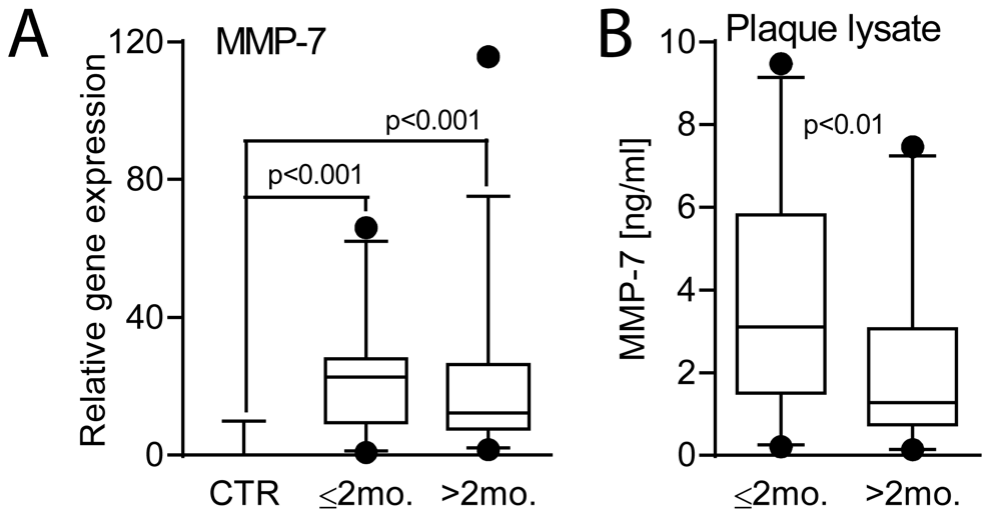
The expression of MMP-7 within atherosclerotic carotid plaques in relation to plaque symptomatology. mRNA (**A**) and protein (**B**) levels of MMP-7 in atherosclerotic carotid plaques were measured in (i) patients with the most recent symptoms (within the last 2 months, n = 35) and (ii) patients with symptoms >2 months ago and asymptomatic patients (>2 months, n = 29). For comparison, mRNA levels of MMP-7 were also analyzed in non-atherosclerotic vessels (common iliac artery) from 10 organ donors (CTR). mRNA levels were quantified by real-time RT-PCR. The expression of β-actin was used as endogenous control. Protein levels of MMP-7 were measured in plaque lysates by EIA. Data are shown as a box and whisker plot with median (Q1, Q3) in the box and the whiskers representing the 5 and 95 percentile in relation to β -actin expression.

In a subgroup of our patients, plaques removed at endarterectomy were available (i.e., 35 patients with clinical symptoms within 2 months and in 28 patients with symptoms more than 2 months prior to collection or with no symptoms). For comparison, mRNA levels of MMP-7 were also analyzed in non-atherosclerotic vessels (common iliac artery) from 10 organ donors. As shown in Fig. 2A, carotid atherosclerotic lesions had markedly higher mRNA levels of MMP-7 than in non-atherosclerotic vessels, with particularly high levels in patients with the most recent symptoms. Indeed, at the protein levels, analyzing MMP-7 levels in plaque lysate by EIA, the patients with the most recent symptoms (i.e., within 2 months) had significantly higher MMP-7 levels than the other patients with concentrations comparable to plasma levels in these patients (Fig. 2B). In contrast, transcript of MMP-7 showed very low expression in PBMC from both patients with symptoms within the last 2 months (n = 16) and healthy controls (n = 16), potentially suggesting that the atherosclerotic lesion itself could have contributed to high plasma levels of MMP-7 in these patients.

### Immunostaining of Carotid Plaques

**Figure 3:**
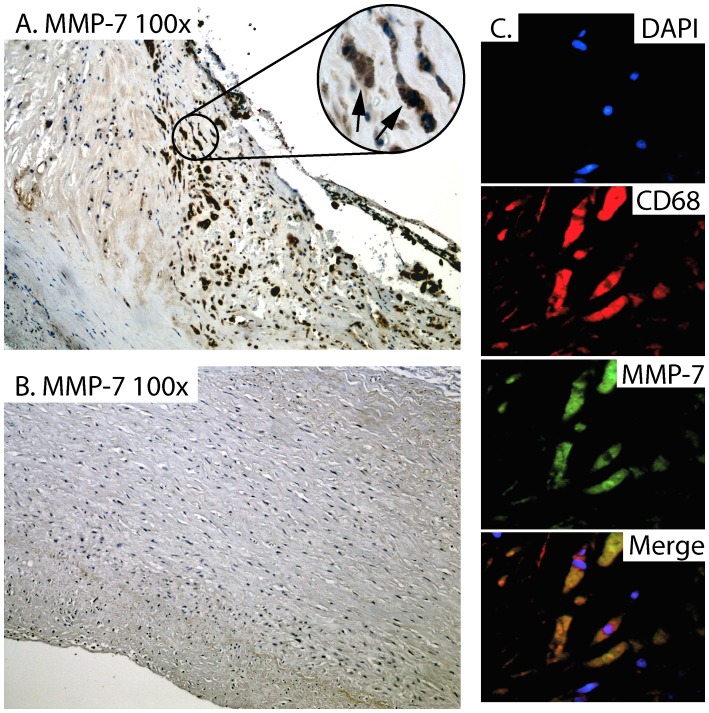
Immunostaining of MMP-7 within atherosclerotic and non-atherosclerotic vessels. Immuhistochemistry of MMP-7 in carotid atherosclerotic plaques (n = 8, symptoms within the recent 2 months) shows strong immunostaining. Representative images obtained with 100× **A** and 400× magnification (highlighted, with arrows on positive cells). Panel **B** shows no or weak immunostaining of MMP-7 in non-atherosclerotic carotid artery obtained from autopsies (n = 5). Panel **C** shows double immunofluorescent staining of MMP-7 (green fluorescence), CD68 (macrophages, red fluorescence) and nucleus (DAPI, blue fluorescence) from carotid atherosclerotic plaques (n = 4). The lower right panel is a merge of the three pictures.

**Figure 4:**
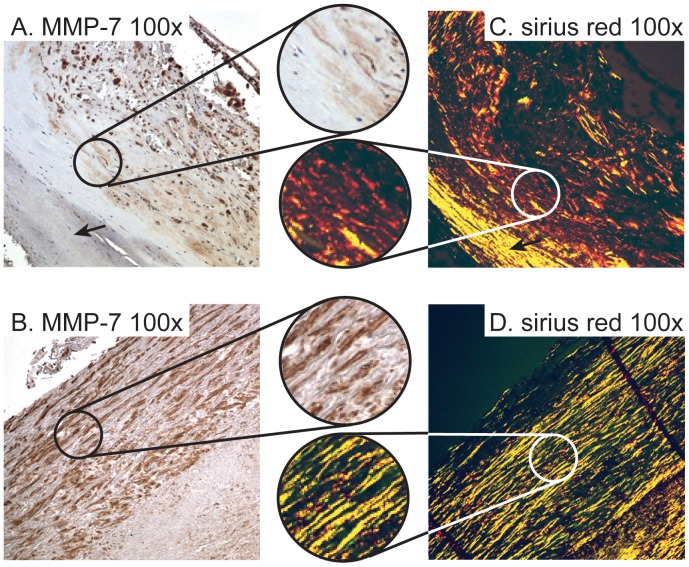
MMP7 staining in areas with thinner and less organized collagen fibers. Immunohistochemical staining of MMP7 in human atherosclerotic plaques at 100× magnification **A** and **B.** Sirius Red staining of collagen in corresponding sections of human atherosclerotic plaques are shown in **C** and **D**. The highlighted sections are from corresponding areas of sequential sections of the same plaques stained with MMP7 and Sirius Red, respectively, at 250× magnification. The arrows indicate area with no positive staining for MMP7 corresponding to areas with more organized and tightly packed collagen fibers.

In the patient group as a whole, mRNA levels of MMP-7 were significantly correlated with mRNA levels of CD68 as a marker of macrophages infiltration (r = 0.40; p<0.05), but not with the pan-leukocyte marker CD45 (r = 0.18, p = 0.20). Indeed, strong immunostaining was shown in sections from patients with symptomatic carotid atherosclerosis (n = 8, symptoms within the recent 2 months) (Fig. 3A), but not in non-atherosclerotic carotid arteries (n = 5) (Fig. 3B), localized to macrophages as shown by immunofluorecent staining (Fig. 3C). MMP-7 may promote matrix degradation, and when performing picrosirius red staining of collagen and conventional immunohistochemical staining of MMP-7 in sequential sections from carotid atherosclerotic plaques (n = 8), we show the presence of MMP-7 predominantly in areas with less organized collagen fibers (Figure 4).

### Regulation of MMP-7 in Primary Monocytes

**Figure 5:**
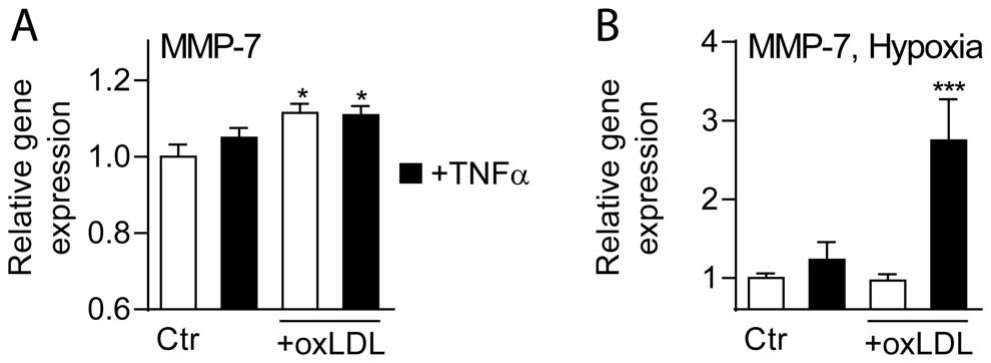
The regulation of MMP-7 expression in primary monocytes. Panel **A** shows the effect of oxLDL (20 µg/ml), TNFα (5 ng/ml) or a combination thereof, panel **B** shows the effect of hypoxia with or without co-stimulation with oxLDL (20 mg/ml), TNFα (5 ng/ml) or a combination thereof. The cells were cultured for 48 hours before experimental starts and cell pellets were harvested 18 hours thereafter. mRNA levels of MMP-7 were quantified by real-time RT-PCR in relation to the expression of the endogenous control gene β-actin. Data are mean±SEM (n = 6) and are given in relation to cells that received vehicle (Ctr) or were cultured in normoxic condition. *p<0.05 versus Ctr (panel **A**). ***p<0.001 versus all other conditions (panel **B**).

Our findings so far suggest markedly up-regulated MMP-7 within atherosclerotic carotid plaques, primary located to macrophages. To further elucidate the regulation of MMP-7 in these cells, we examined the expression of MMP-7 (mRNA) in primary monocytes, obtained from 7 healthy blood donors, after culturing for 48 hours before further culturing for 18 hours with and without stimulation with oxLDL (20 µg/mL), TNFα (5 ng/mL) or a combination thereof with and without hypoxia (see Methods). As depicted in Fig. 5A, oxLDL increased MMP-7 levels in primary monocytes without any additional effect of TNFα co-incubation. While this MMP-7-inducing effect of oxLDL was abrogated during hypoxia, the combination of oxLDL, TNFα and hypoxia markedly induced MMP-7 expression in these cells (Fig. 5B). The high expression in monocytes that had been activated for 18 hours after culturing for 48 hours compared with the nearly undetectable mRNA levels of MMP-7 in freshly isolated PMBC (see above) suggest that some degree of differentiation and activation is need to induce MMP-7 transcription in these cells.

### Plasma Levels of MMP-7 are Associated with Total Mortality in Patients with Carotid Atherosclerosis

**Figure 6:**
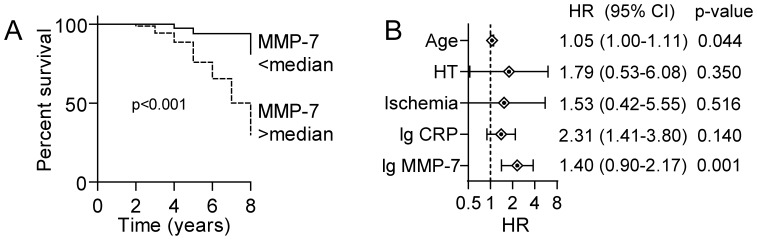
Association between plasma levels of MMP-7 and adverse outcome in patients with carotid atherosclerosis. Panel **A** shows Kaplan–Meier curve with the cumulative incidence of all-cause mortality during the entire study (mean follow-up 3.5 years) according to dichotomized MMP-7 levels (Cut-off median: 1.96 ng/mL). Panel **B** shows multi-variable analyses of predictors of all-cause mortality (direct entry). CRP and MMP-7 show expressed per SD change.

Our findings show up-regulated MMP-7 levels in patients with carotid atherosclerosis, in particularly in those with the most recent symptoms, potentially related to the interaction between inflammation, modified lipids and ischemia/hypoxia within plaque monocytes/macrophages. During follow-up (mean follow-up time 3.5 years), 29 of the patients that were included in the present study died, but except for these patients, none of the patients were lost of other reasons during follow-up. The majority (n = 14) of the mortalities were caused by cerebrovascular or cardiovascular events, 9 patients died of cancer and the other 6 patients died of various other causes (2 died of chronic obstructive pulmonary disease exacerbation, 1 died of liver failure, 1 died of amyloidosis, 1 died of interstitial lung disease with fibrosis and 1 died a sudden death without specific cause of death). As depicted in Fig. 6A, high MMP-7 levels (i.e., >median) at baseline were significantly associated with adverse outcome as shown by Kaplan Meier curves. There were no differences in MMP-7 levels at baseline between those who died of cardiovascular/cerebrovascular events and those who died of other causes including those who died of cancer (2.96 [1.25–9.29] ng/mL versus 2.76 [1.57–9.14] ng/mL [median and ranges], cerebro/cardiovascular and other causes of mortality, respectively; p = 0.81). High sensitivity CRP is regarded as a reliable marker of inflammation and raised CRP levels have been associated with mortality in several studies of cardiovascular diseases [17]. In the present study, both MMP-7 and CRP (as assessed by a high-sensitivity assay) were associated with all-cause mortality in univariate cox regression. However, when evaluated together in multivariable analysis with other predictors (age, hypertension, ischemia on MRI) imbalanced between survivors and non-survivors, MMP-7 was together with age, the only significant predictors of mortality, with the strongest association with MMP-7 (Fig. 6B). Although MMP7 and CRP were correlated, the association was relatively weak (r = 0.15) explaining only approx. 4% of the variation in each other. Thus, it is likely that MMP7 may reflect other mechanisms more closely related to adverse outcome than CRP in this population.

## Discussion

MMP-7 together with other MMPs has previously shown to be elevated in patients with atherosclerosis [7]. Pelisek et al. also reported increased serum MMP-1 and MMP-7 levels in 64 patients with histological unstable atherosclerotic carotid lesions [7]. By using a microarray approach, Razuvaev et al. examined mRNA levels in 106 carotid plaques, and showed that high expression of MMP-7 and MMP-9 was associated with an unstable plaque phenotype [18]. In the present study we extend these previous findings by showing increased MMP-7 levels systemically as well as within the atherosclerotic lesion in patients with carotid atherosclerosis with the most recent symptoms (i.e., within two months). Moreover, we demonstrate that MMP-7 within the lesion is primarily located to macrophages, potentially reflecting the combined action of inflammation, modified lipids and hypoxia. Notably, MMP-7 was predominantly found in areas with less organized collagen fibers suggesting a possible influence of MMP-7 on collagen structure and strength. Finally, we report that high plasma levels of MMP-7 in patients with carotid atherosclerosis are independently associated with mortality during follow-up. Our findings further support a link between MMP-7 and plaque instability in patients with carotid atherosclerosis, potentially reflecting macrophage and matrix degrading mechanisms.

A major finding in the present study was the association between plasma levels of MMP-7 and plaque symptomatology as well as total mortally during follow-up. In fact, MMP-7 was the strongest predictor for adverse outcome followed by age. The ability to predict outcome in patients with carotid plaque is an important challenge, and our findings may suggest that MMP-7 should be further investigated as a biomarker in these patients. The strength of a biomarker often reflects its ability to mirror up-stream pathogenic pathways, and our findings suggest that this also could be the case for MMP-7. Thus, we found that the atherosclerotic carotid plaque contained large amount of MMP-7, and it is possible that plasma levels of MMP-7, at least to some degree, could reflect MMP-7 levels within the lesion. Moreover, our data suggest that MMP-7 within the carotid plaques is primarily located to macrophages, and notably, several pro-atherogenic stimuli were shown to enhance MMP-7 expression in these cells. Hence, the combined stimulation of inflammatory mediators (i.e., TNFα), oxLDL and hypoxia, that are seen during ischemia, were found to markedly increase MMP-7 expression in monocytes. It is possible that the association between MMP-7 in plasma and recent plaque symptomatology as well as mortality may reflect its relation to several up-streams pathogenic pathways that are involved in plaque destabilization. The link between MMP-7, macrophages and plaque rupture has also been supported by Halpert et al., reporting that MMP-7 was expressed by lipid laden macrophages at sites of plaque rupture in atherosclerotic carotid lesions [10].

Even if the present study did not examine in what way MMP-7 could promote plaque destabilization, some studies suggest that MMP-7 is not only a marker but also a mediator during plaque rupture. In atherosclerosis, MMP-7 has been linked to apoptosis of vascular SMC. Williams et al. demonstrated that MMP-7 is involved in the cleavage of N-cadherin, a cell-cell junction protein in vascular SMCs, and enhances apoptosis of these cells, contributing to plaque rupture [19]. Indeed, these authors reported markedly decreased apoptosis in atherosclerotic lesions from MMP-7 deficient mice [19]. MMP-7 produced by activated macrophages has a potent capacity to degrade numerous matrix components including proteoglycans, insoluble elastin and fibronectin. In relation to atherosclerosis, Halpert et al. reported that MMP-7 is more effective than other MMPs in degrading versican, a proteoglycane usually found at rupture prone areas in atherosclerotic lesion [10]. In the present study we showed the presence of MMP-7 predominantly in areas with less organized collagen fibers. This may suggest a possible influence of MMP-7 on collagen structure and strength, further supporting a possible role for MMP-7 in plaque destabilization.

The present study has some limitations such as a relatively small study population and a relatively low number of adverse events (death) during follow-up. We also lack information on specific cardiovascular and cerebrovascular diagnosis in relation to mortality. Although we were primarily interested in large differences between patients and controls, the number of controls could have been higher and the patients and controls could also have been more adequately matched. Nonetheless, our findings suggest that MMP-7 could contribute to plaque instability in patients with carotid atherosclerosis, potentially involving macrophage-related mechanisms. Our findings also suggest that MMP-7 should be further investigated as a biomarker in these patients including studies in larger study population with more long time follow-up.
